# Genome-Wide Analysis of the PYL Gene Family in *Betula platyphylla* and Its Responses to Abiotic Stresses

**DOI:** 10.3390/ijms252413728

**Published:** 2024-12-23

**Authors:** Jiajie Yu, Ruiqi Wang, Xiang Zhang, Su Chen

**Affiliations:** 1 School of Agriculture, Liaodong University, Dandong 118003, China; 13514554189@163.com; 2 State Key Laboratory of Tree Genetics and Breeding, Northeast Forestry University, Harbin 150040, China; yjswrq@outlook.com

**Keywords:** PYL gene family, birch (*Betula platyphylla*), ABA signaling, bioinformatic analysis, abiotic stress response

## Abstract

Abscisic acid (ABA) is a key phytohormone that participates in various plant biological processes, such as seed germination, senescence, stomatal movement, and flowering. In the ABA signal transduction pathway, Pyrabactin Resistance 1 (PYR1)/PYR1-Like (PYL)/Regulatory Component is the core module for ABA perception. In this study, a total of 12 PYL family members were identified in birch (*Betula platyphylla* Suk.) from a genome-wide range that can be divided into 3 subgroups according to their evolutionary relationships. The physiochemical properties of the 12 *BpPYL*s were characterized, and the members of the same subgroups share more similar exon–intron and motif patterns. The results of synteny analysis showed two syntenic gene pairs within BpPYL family members and 12, 8, 19, and 6 syntenic gene pairs between *BpPYL*s and *AtPYL*s, *OsPYL*s, *PtPYL*s, and *ZmPYL*s, respectively. Multiple *cis*-acting elements were identified in the promoters of *BpPYL*s, including stress response, phytohormone signaling, and growth and development. The results of GO and KEGG enrichment analysis showed that *BpPYL*s were enriched in the pathways mainly related to ABA signaling and cell communication. The results of RT-qPCR verified the expressional responses of *BpPYL*s to ABA, salt, and PEG treatments. Furthermore, the positive roles of *BpPYL3* and *BpPYL11* were proven by using salt-tolerant yeast transformation. This study provides a reference for research on ABA signal transduction and forest tree responses upon abiotic stresses.

## 1. Introduction

Abscisic acid (ABA) was found for the first time in the 1970s to be involved in fruit abscission and plant dormancy [1]. Over the past decades, it has been proven to participate widely in multiple plant biological processes, including seed germination, cell elongation and division, leaf senescence, stomatal aperture control, photosynthesis, flowering, root growth, and fruit ripening [2,3,4,5,6,7]. Furthermore, ABA plays an important role in the plant abiotic stress response [8,9,10]. In Huang et al.’s study [11], exogenous ABA application enhanced the cold tolerance of bermudagrass by maintaining cell membrane stability and improving photosynthesis. According to Sripinyowanich et al.’s research [12], exogenous ABA increased the survival rate of rice seedlings under salt stress, triggered more proline accumulation, and thus, promoted salt tolerance. ABA was also proven to have an important function in regulating the accumulation of several essential proteins upon a combination of water deficit and heat stress [13]. In birch, ABA increased the freezing resistance of birch cell suspension cultures [14]. In response to abiotic stresses such as drought, salinity, and cold stress, the ABA level in plants tends to show a major change, which activates the expression of related downstream genes [3,15].

Substantial research advancement has been made over the past decade in unveiling the mechanism of ABA signaling transduction, including ABA perception. In plants, ABA is perceived by the ABA receptors [16]. One of the most important and key ABA receptors is the Pyrabactin Resistance 1 (PYR1)/PYR1-Like (PYL)/Regulatory Component [6]. PYLs, phosphatase type 2Cs (PP2Cs), and SUCROSE NONFERMENTING 1 (Snf1)-related protein kinase 2s (SnRK2s) constitute the core module of the ABA signaling pathway [17]. When ABA is at a low level in plants, PP2Cs deactivate SnRK2s, and therefore, lead to the deactivation of downstream genes [18,19,20]. Upon abiotic stress, the PYL bound by ABA goes through a structural change and thereby forms interactions with PP2Cs, which frees the SnRK2s from the inhibition state [21,22,23]. The activated SnRK2s are able to phosphorylate downstream proteins like the ABA-responsive element (ABREs)/abscisic acid binding factor (ABF)/ABI5 clade of bZIP transcription factors, which regulate the physiological responses of plants upon abiotic stresses [18,24,25,26].

With the development of genome sequencing and bioinformatic tools, PYL family members have been identified in various plant species such as tobacco [27], rice [28], soybean [29], tomato [30], wheat [31], maize [32], rapeseed [33], strawberry [34], poplar [35], rubber tree [36], and sunflower [37]. In the model plant *Arabidopsis*, a total of 14 PYL family members have been identified, named *PYR1* and *PYL1–PYL13* [38,39]. They exhibited high conservation in amino acid sequence and were classified into three subgroups according to their phylogenetic relationships [40]. All 14 members contained a START (STAR-RELATED LIPID-TRANSFER) conserved domain, which was marked by a seven-stranded β-sheet surrounded by two α-helices. These structures formed a ligand-binding “pocket”, which is formed by the L2 loop between the α3 helix and β2 strand, the L4 loop between the β3 and β4 strands, and the L5 loop between the β5 and β6 strands, with helix α4 as the entrance [25]. This “pocket” is key for the binding of ABA to PYLs.

Increasing evidence shows that *PYL* genes play pivotal roles in plant responses to abiotic stresses. In the grape [41], the expression levels of the *VaPYL1*, *VaPYL4*, *VaPYL5*, and *VaPYL13* genes were significantly increased upon cold stress. The ectopic expression of *VaPYL4* in tobacco increased the ABA sensitivity as well as the cold tolerance of the transgenic plants. *Arabidopsis* transformed with *VaPYL4* showed improved tolerance to salt and drought stress. Yao et al. identified *StPYL16* via transcriptomic analysis in the potato [42]. This gene is highly responsive to drought stress. The transient and stable expression of *StPYL16* in *Arabidopsis* enhanced the drought stress tolerance of the transgenic plants. At the metabolic level, the transgenic *Arabidopsis* plants showed a lower content of malondialdehyde (MDA) and higher superoxide dismutase (SOD), peroxidase (POD), and catalase (CAT) activities. In *Populus euphratica*, *PePYL4* was found to be responsive to ABA and osmotic and dehydration treatments [43]. The poplar plants overexpressed with this gene exhibited increased ABA sensitivity and reduced stomatal apertures compared with the wild type. Furthermore, the transgenic poplar had higher water use efficiency, higher photosynthetic activity, lower transpiration, and more accumulated biomass. 

With the growing demand for food security and ecological protection, the need to understand plant responses to abiotic stress, such as cold, drought, salt, and heavy metal stress, has led to increasing research interest. It is meaningful and urgent to investigate the related molecular pathways and to explore potential gene resources for plant genetic breeding. White birch (*Betula platyphylla* Suk.), a widely distributed forest tree species in the temperate and frigid zones, provides valuable sources in the food, medication, industry, and ecology fields. Furthermore, birch shows outstanding tolerance to abiotic stresses, which makes it an ideal research subject for plant abiotic stress response [44]. In this study, a total of 12 PYL family members were identified in birch from a genome-wide range. The physiochemical properties, phylogenetic relationships, exon–intron patterns, *cis*-acting elements, and enriched pathways were investigated by using bioinformatic tools. The expressional responses of *BpPYL*s to multiple abiotic stresses were characterized by using RT-qPCR. The positive roles of *BpPYL3* and *BpPYL11* in salt tolerance were proven with the salt-tolerant yeast transformation method. This study lays a foundation for research on ABA perception pathways and the forest tree stress response mechanism.

## 2. Results

### 2.1. Identification, Chromosome Localization, and Physiochemical Properties of PYL Genes in Birch

**Figure 1:**
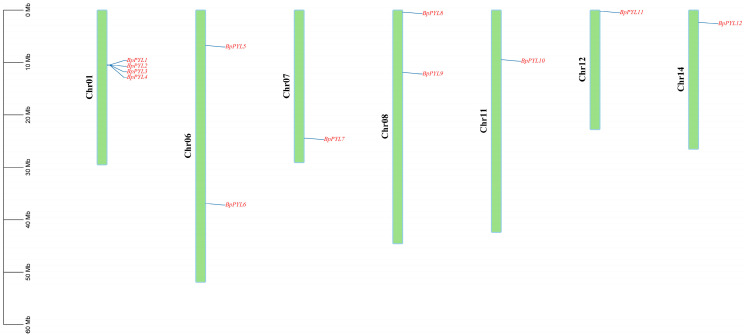
Chromosome location of *BpPYL*s. Green stripes represent birch chromosomes. The scale bar on the left measures the chromosome length (Mb).

To identify all the PYL family members in birch within the whole genome range, the coding sequences (CDSs) and the amino acid sequences of *PYL*s in *Arabidopsis* served as the queries to probe the potential *BpPYL*s throughout the birch genome data. After carrying out BLASTp and Hidden Markov Model (PF10604) searches, the results of InterPro (IPR019587) and CDD screening showed that a total of 12 PYL family members were identified in birch. The 12 *BpPYL*s were distributed unevenly on 7 chromosomes (Figure 1). The largest quantity of *BpPYL*s (*BpPYL1*, *BpPYL2*, *BpPYL3*, *BpPYL4*) were distributed on Chromosome 1 (Chr01). Only one or two *BpPYL*s were distributed on one of the other chromosomes.

To further investigate the basic physiochemical properties of the 12 *BpPYL*s, their genomic sequence lengths, amino acid numbers, molecular weights, etc. were analyzed, and the results are shown in Table 1. The amino acid numbers of the BpPYLs ranged from 129 to 296 aa and the molecular weights from 14,432.06 to 32,794.00. The isoelectric points indicated the BpPYLs carry negative charges. The cellular localization prediction showed that most of the BpPYLs were localized in the cytoplasm, with BpPYL2 and BpPYL5 in the chloroplast, BpPYL3 and BpPYL7 in both the cytoplasm and chloroplast, and BpPYL8 and BpPYL11 in the chloroplast and nucleus.

### 2.2. Phylogenetic Relationships and Gene Structures of BpPYLs

**Figure 2:**
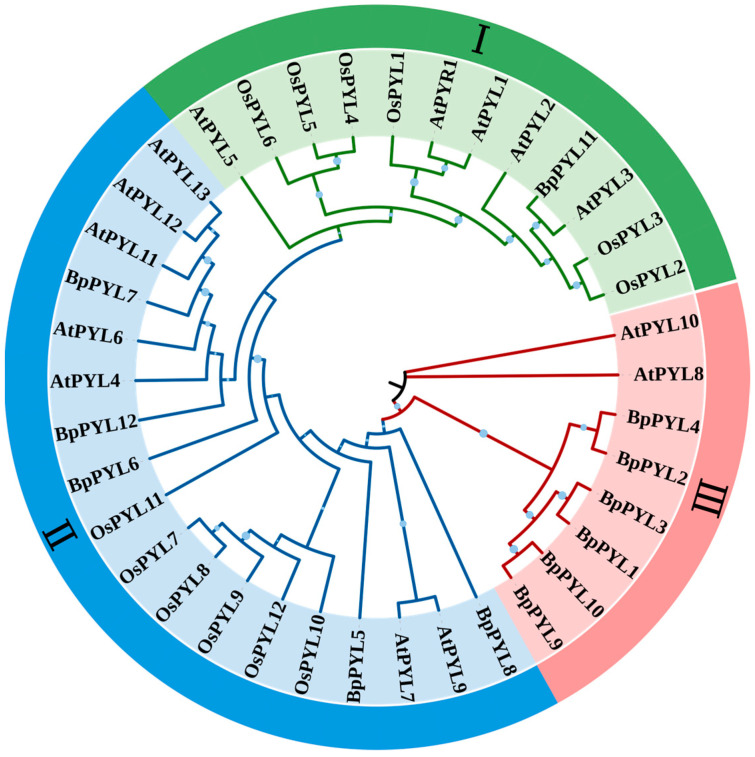
Phylogenetic relationships of BpPYLs, AtPYLs, and OsPYLs. The phylogenetic tree (1000 bootstrap replicates) was constructed using MEGA7.0.

The phylogenetic relationships between the BpPYLs were investigated by constructing a phylogenetic tree with PYL family members from birch, *Arabidopsis* (*Arabidopsis thaliana*), and rice (*Oryza sativa*). The 12 BpPYLs, 14 AtPYLs, and 12 OsPYLs were clustered into 3 subgroups (Ⅰ, Ⅱ, and Ⅲ) (Figure 2). In Subgroup Ⅰ, there were five AtPYLs, six OsPYLs, and only one BpPYL, which was BpPYL11. In Subgroup Ⅱ, there were seven AtPYLs, six OsPYLs, and five BpPYLs. In Subgroup Ⅲ, there were two AtPYLs and six BpPYLs. 

**Figure 3:**
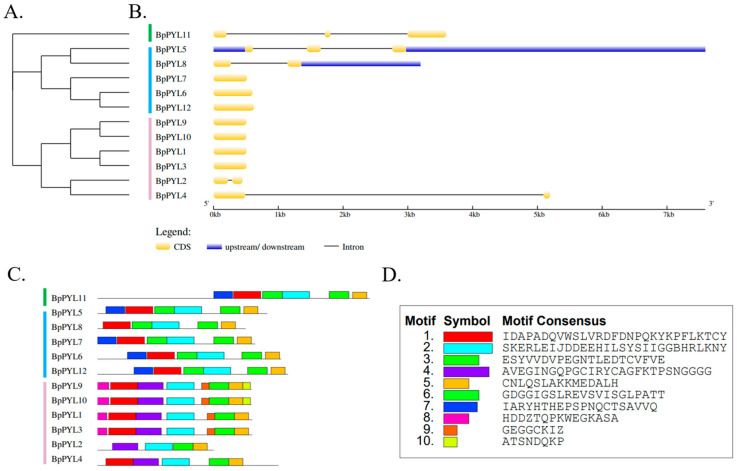
Exon–intron and motif patterns of *BpPYL*s. (**A**) Phylogenetic tree of *BpPYL*s. (**B**) Exon–intron pattern of *BpPYL*s. Yellow box represents CDS. Blue box represents untranslated region (UTR). Line represents intron. The scale bar measures the length (bp) of genes in kilo base pairs (Kb). (**C**) Motif patterns of BpPYLs. (**D**) Amino acid sequence of the motifs.

To further investigate the sequence characteristics of the *BpPYL*s, gene structures and motif patterns were analyzed by using the online tools GSDS2.0 and MEME. The results were visualized and displayed in the manner of phylogenetic relationships (Figure 3A). The *BpPYL* members that have closer phylogenetic relationships share more similarities in the exon–intron and motif patterns (Figure 3B,C). For instance, *BpPYL11* has relatively distant evolutionary relationships with the other family members. This gene also has unique exon–intron and motif patterns, with no other *BpPYL*s alike. *BpPYL1*, *BpPYL9*, and *BpPYL10* have relatively close evolutionary relationships in Subgroup Ⅲ. They share relatively high similarities in exon–intron and motif patterns. The motif sequences are listed in Table S1.

### 2.3. Syntenic Relationships of BpPYLs

**Figure 4:**
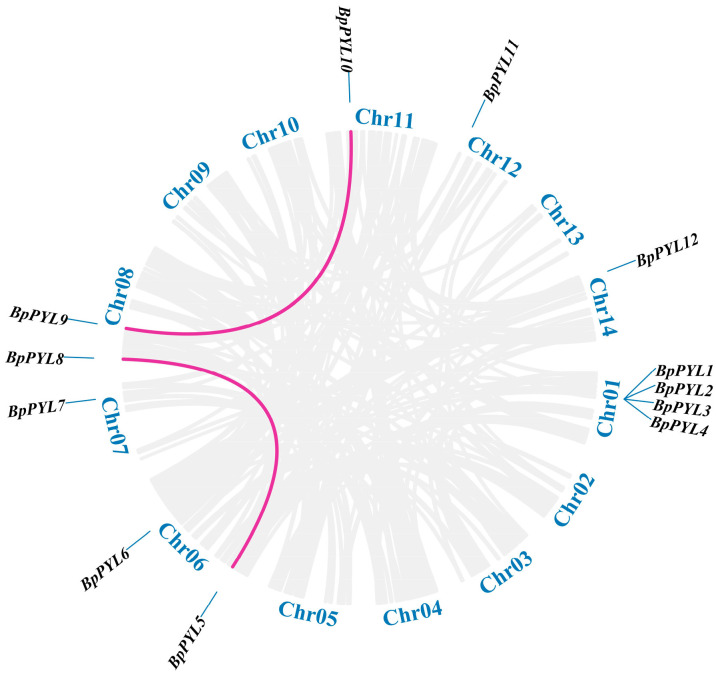
Syntenic relationships between *BpPYL*s. Pink lines represent syntenic gene pairs. The chromosome number is shown in blue.

To investigate the gene duplication events in the evolution process of *BpPYL*s, the intraspecific syntenic relationships between the *BpPYL*s were analyzed and visualized (Figure 4). Two syntenic gene pairs, *BpPYL9* and *BpPYL10* along with *BpPYL5* and *BpPYL8*, were identified. Ka symbolizes synonymous mutation and Ks symbolizes non-synonymous mutation. The Ka/Ks ratio of *BpPYL5*/*BpPYL8* was less than 1, which indicates purifying selection (Table 2). The Ka and Ks of *BpPYL9*/*BpPYL10* were both zero, which indicates gene segment insertion or deletion during the evolution.

**Figure 5:**
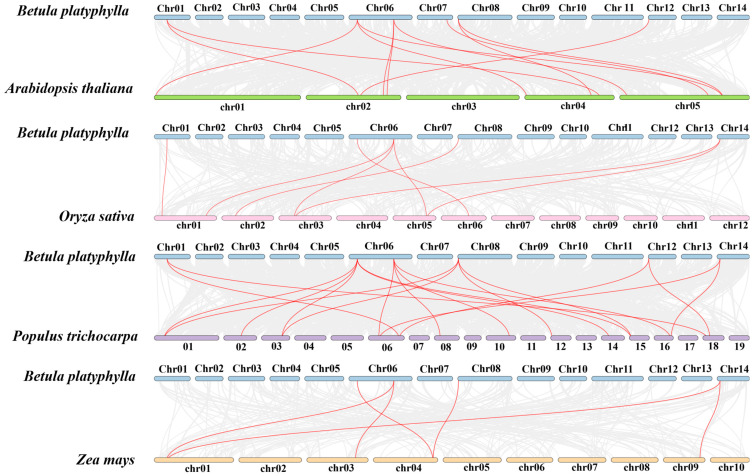
Syntenic relationships between *BpPYL*s and *OsPYL*s, *PtPYL*s, and *ZmPYL*s, respectively. Red lines represent syntenic gene pairs. Grey lines in the background represent collinear blocks. The chromosome number is shown beside each chromosome.

To further identify the evolutionary relationships of PYL members from birch with those of *Arabidopsis*, rice, poplar, and maize, the syntenic gene pairs between *BpPYL*s and *AtPYL*s, *OsPYL*s, *PtPYL*s, and *ZmPYL*s, respectively, were investigated and visualized (Figure 5). The syntenic gene pair numbers between the *BpPYL*s and *AtPYL*s, *OsPYL*s, *PtPYL*s, and *ZmPYL*s, respectively, were 12, 8, 19, and 6. The largest number of birch genes that had syntenic relationships with those in the other species are distributed on Chr06 and Chr08. The detailed information of these syntenic gene pairs is listed in Tables S2–S5.

### 2.4. Cis-Acting Elements in the Promoters of BpPYLs

**Figure 6:**
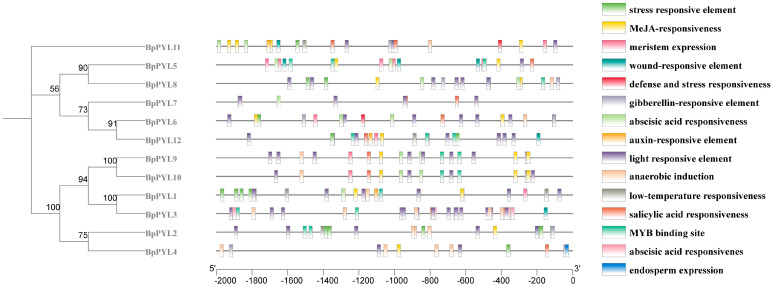
The *cis*-acting elements in the promoters of *BpPYL*s. Different colored boxes represent different *cis*-acting elements.

The distribution of different *cis*-acting elements in the promoters reflects the gene function. In this case, the *cis*-acting elements in the promoters of the *BpPYL*s were analyzed by the online tool PlantCARE (Figure 6). These *cis*-acting elements can be classified mainly into three categories, including stress response (such as stress responsiveness, wound responsiveness, low-temperature responsiveness, salicylic acid responsiveness), phytohormone signaling (such as MeJA responsiveness, gibberellin responsiveness, abscisic acid responsiveness, auxin responsiveness), and growth and development (meristem expression, light responsiveness, anaerobic induction, endosperm expression). Almost all the *BpPYL* promoters contain stress responsive and abscisic acid responsiveness elements. The promoters of *BpPYL1*, *BpPYL2*, *BpPYL11* and *BpPYL12* had low-temperature responsiveness elements. It suggests the potential important roles of *BpPYL*s in stress response and ABA signaling. The location sites of these *cis*-acting elements were listed in Table S6.

### 2.5. Enriched Pathways of BpPYLs Functioning

**Figure 7:**
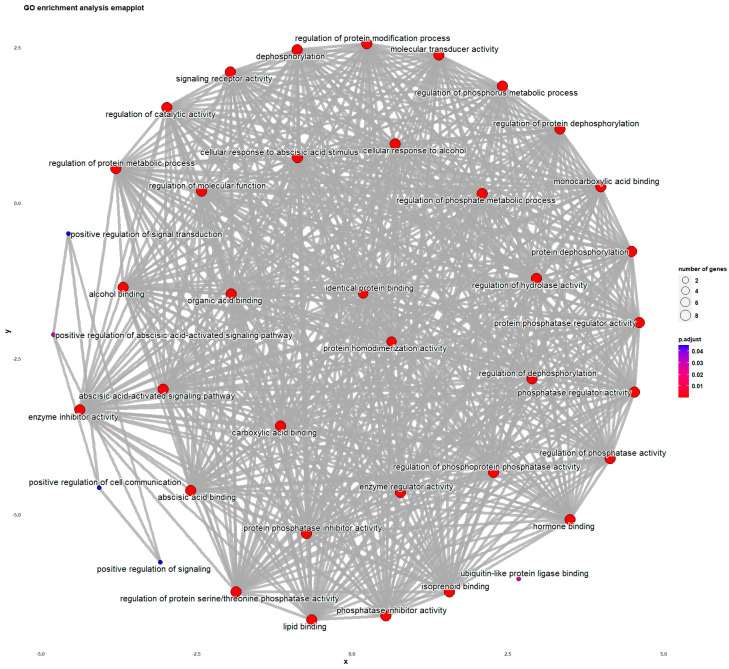
GO enrichment analysis of *BpPYL*s. Red dots represent biological pathways.

**Figure 8:**
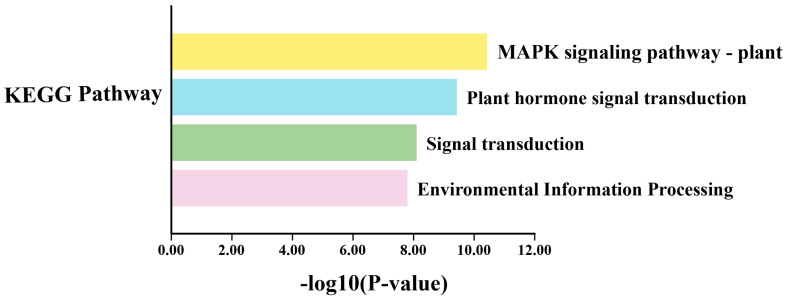
KEGG pathway enrichment analysis of *BpPYL*s.

GO (gene ontology) enrichment and KEGG (Kyoto Encyclopedia of Genes and Genomes) analyses were conducted to investigate the pathways in which *BpPYL*s may be involved. The result of GO enrichment (Figure 7) showed various biological processes, including positive regulation of the abscisic acid-activated signaling pathway, positive regulation of signal transduction, positive regulation of cell communication, and positive regulation of signaling. The result of KEGG analysis (Figure 8) showed the enrichment of metabolic pathways including the MAPK signaling pathway and plant hormone signal transduction.

### 2.6. Tissue-Specific Expression Patterns of BpPYLs

**Figure 9:**
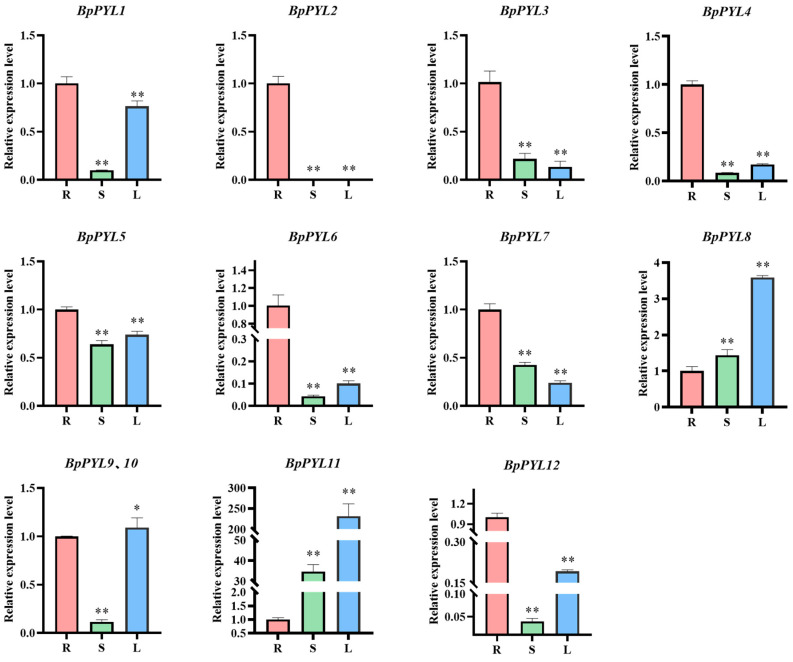
Tissue-specific expression patterns of *BpPYL*s. The tissue-specific expression analysis was performed by using RT-qPCR with the birch root, stem, and leaf as templates. R: root; S: stem; L: leaf. There were three biological and three technical replications. The expression level of each gene in the root was normalized to 1.0, and the relative expression levels were calculated using the 2^−ΔΔCt^ method (*t*-test, * *p* < 0.05, ** *p* < 0.01).

To investigate the expression patterns of *BpPYL*s in different birch tissues, RT-qPCR was performed with the RNA of the birch root, stem, and leaf as templates. As shown in Figure 9, most (8/12) of the *BpPYL*s exhibited the highest expression levels in the root. Furthermore, most (6/8) of these 8 genes exhibited higher expression levels in the leaf than in the stem. For instance, the expression level of BpPYL2 in the root was 4.65 times that in the stem and 7.46 times that in the leaf. The expression level of BpPYL7 in the root was 2.35 times that in the stem and 4.13 times that in the leaf. There were exceptions; *BpPYL8*, *BpPYL11*, *BpPYL9*, and *BpPYL10* showed the highest expression levels in the leaf. The expression level of BpPYL8 in the leaf was 3.59 times that in the root and 2.49 times that in the stem. The related RT-qPCR data are listed in Table S7.

### 2.7. Expression Patterns of BpPYLs Under ABA Treatment

**Figure 10:**
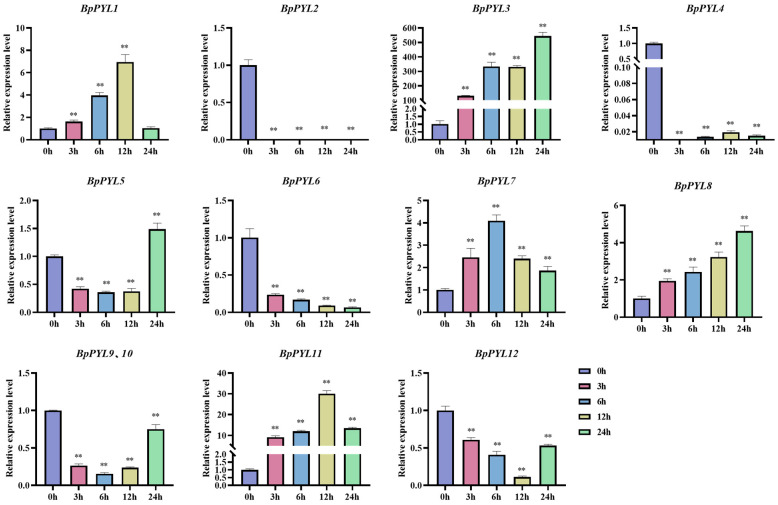
Expressional responses of *BpPYL*s upon ABA treatment. The expression analysis was performed by using RT-qPCR with birch roots as templates. There were three biological and three technical replications. The concentration of ABA treatment was 100 µM, and the sampling time points were 0, 3, 6, 12, and 24 h after treatment. The expression level of each gene at 0 h was normalized to 1.0, and the relative expression levels were calculated using the 2^−ΔΔCt^ method (*t*-test, ** *p* < 0.01).

To explore the responses of *BpPYL*s upon ABA treatment, the relative expression levels of *BpPYL*s in birch roots were quantified by using RT-qPCR after different times (0 h, 3 h, 6 h, 12 h, 24 h) of ABA treatment (Figure 10). The results showed that all the *BpPYL*s were responsive to ABA treatment to different degrees and in different patterns. The relative expression levels of some *BpPYL*s (*BpPYL1*, *BpPYL7*, and *BpPYL11*) exhibited a low-high-low trend, while the highest expression level appeared at different time points. The relative expression levels of some *BpPYL*s (*BpPYL5*, *BpPYL9*, *BpPYL10*, and *BpPYL12*) exhibited a high-low-high trend, while the lowest expression level appeared at different time points. There were also some genes that were upregulated (*BpPYL3*, *BpPYL8*) or downregulated (*BpPYL6*) throughout. There were exceptions; the expression levels of *BpPYL2* and *BpPYL4* were decreased by ABA treatment to significantly low levels. Otherwise, the genes that exhibited similar expressional trends may show differential degrees of expressional change. For instance, the *BpPYL1* expression was the highest at 12 h, approximately 6 times that of the 0 h time point, while the *BpPYL11* expression at 12 h was approximately 30 times that of the 0 h time point. The related RT-qPCR data are listed in Table S8.

### 2.8. Expression Patterns of BpPYLs Under Salt Treatment

**Figure 11:**
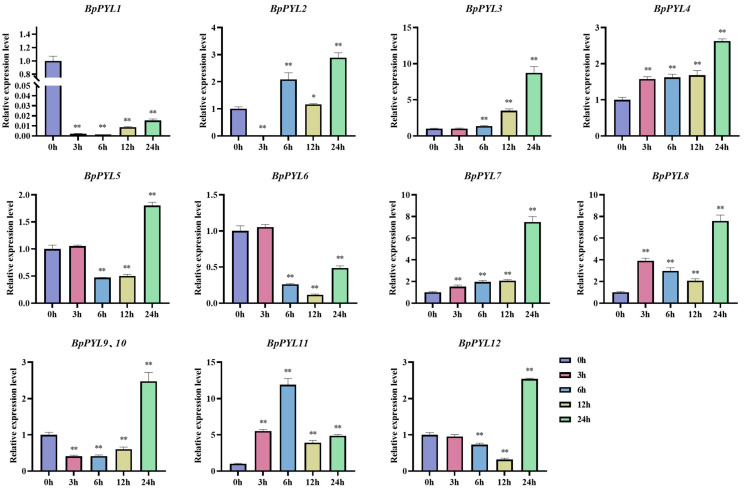
Expressional response of *BpPYL*s upon salt treatment. The expression analysis was performed by using RT-qPCR with birch roots as templates. There were three biological and three technical replications. The concentration of salt treatment was 200 mM, and the sampling time points were 0, 3, 6, 12, and 24 h after the treatment. The expression level of each gene at 0 h was normalized to 1.0, and relative expression levels were calculated using the 2^−ΔΔCt^ method (*t*-test, * *p* < 0.05, ** *p* < 0.01).

To explore the responses of *BpPYL*s upon salt treatment, the relative expression levels of *BpPYL*s in birch roots were quantified by using RT-qPCR after different times (0 h, 3 h, 6 h, 12 h, 24 h) of salt treatment (Figure 11). All the *BpPYL*s were responsive to salt treatment to different degrees and in different patterns. After 3 or 6 h of salt treatment, there were seven *BpPYL*s (*BpPYL1*, *BpPYL2*, *BpPYL5*, *BpPYL6*, *BpPYL9*, *BpPYL10*, and *BpPYL12*) whose expression levels exhibited a decrease trend. The most significant decrease trends were shown in *BpPYL1* and *BpPYL2*, reaching at 3 h approximately 0.002 and 0.008 times the level at 0 h, respectively. The other five *BpPYL*s, which showed upregulation after 3 or 6 h of salt treatment, also exhibited different expression patterns. For instance, the expression levels of *BpPYL3* and *BpPYL4* kept increasing throughout, while the expression level of *BpPYL11* exhibited an increase trend until 6 h and then a decrease trend. The related RT-qPCR data are listed in Table S9.

### 2.9. Expression Patterns of BpPYLs Under PEG Treatment

**Figure 12:**
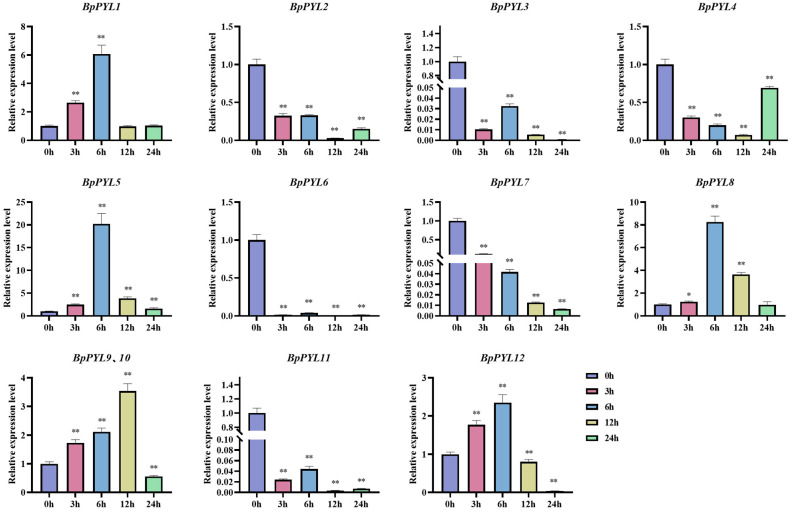
Expressional response of *BpPYL*s upon PEG treatment. The expression analysis was performed by using RT-qPCR with birch roots as templates. There were three biological and three technical replications. The concentration of PEG treatment was 20% (*W*/*V*), and sampling time points were 0, 3, 6, 12, and 24 h after the treatment. The expression level of each gene at 0 h was normalized to 1.0, and relative expression levels were calculated using the 2^−ΔΔCt^ method (*t*-test, * *p* < 0.05, ** *p* < 0.01).

To explore the responses of *BpPYL*s upon PEG treatment, the relative expression levels of *BpPYL*s in birch roots were quantified by using RT-qPCR after different times (0 h, 3 h, 6 h, 12 h, 24 h) of PEG treatment (Figure 12). Half of the *BpPYL*s (*BpPYL2*, *BpPYL3*, *BpPYL4*, *BpPYL6*, *BpPYL7*, and *BpPYL11*) were downregulated upon PEG treatment to different degrees. The relative expression levels of *BpPYL3* and *BpPYL6* at 3 h were approximately 0.010 and 0.015 times those at 0 h, respectively. The relative expression levels of the *BpPYL*s that were upregulated upon PEG treatment showed an increase-peak-decrease trend. The peaks appeared at 6 h or 12 h. For instance, the expression level of *BpPYL5* at 6 h was approximately 20.24 times that at 0 h. The related RT-qPCR data are listed in Table S10.

### 2.10. Positive Functions of BpPYL3 and BpPYL11 in Salt Tolerance

**Figure 13:**
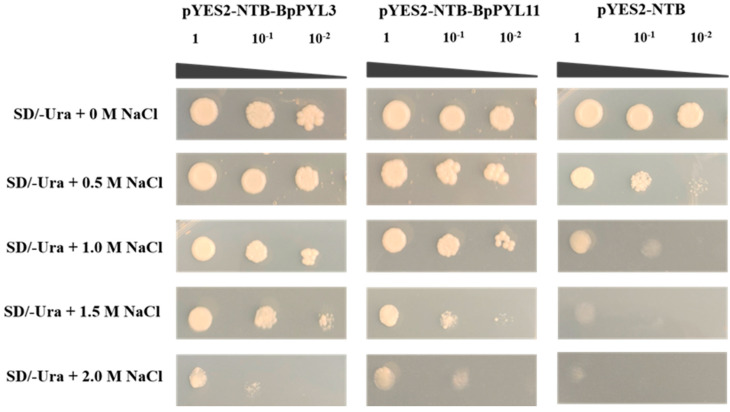
Functions of *BpPYL3* and *BpPYL11* in salt tolerance. Negative control is pYES2-NTB. SD means nutrition-deprived yeast medium, and 1, 10^−1^, 10^−2^ indicate different dilutions of yeast fluids.

According to the expressional responses of *BpPYL*s upon salt treatment, *BpPYL3* and *BpPYL11* were selected as representatives for the verification of their functions in salt tolerance. A salt-resistant yeast transformation assay was conducted to prove the influences of these two genes on the salt tolerance of yeast (Figure 13). The yeast transformed with either pYES2-NTB-BpPYL3 or pYES2-NTB-BpPYL11, and the pYES2-NTB (negative control), were able to grow on the media with no NaCl added, which indicated successful transformation. With the increase in NaCl concentration in the yeast media, the yeast transformed with pYES2-NTB-BpPYL3 or pYES2-NTB-BpPYL11 were still able to grow, while the negative control was not. The results proved the positive functions of *BpPYL3* and *BpPYL11* in salt tolerance.

## 3. Discussion

Since ABA was found for the first time in the 1970s to play important roles in fruit abscission and plant dormancy [1], the wide range of ABA functions and intricate ABA signaling pathways have aroused increasing interest in various kinds of plant research. The PYL receptor is the core module of ABA signaling. To date, *PYL* genes have been identified in various plant species. For instance, a total of 13 PYLs were identified in rice. These members are responsive to multiple abiotic stresses (drought, ABA, salinity, and temperature) to different degrees [28]. In the sunflower, a total of 19 PYL members were identified. Most of these members are responsive to ABA treatment. Half of these members were upregulated upon PEG6000 and salt treatments [37]. However, the PYL family members in birch, a forest tree species with good stress tolerance, remain to be identified and characterized.

In this study, a total of 12 PYL family members were identified in the birch genome and named *BpPYL1* to -*12*. These *BpPYL*s were classified into three subgroups according to their phylogenetic relationships. In Subgroup Ⅰ, there is only one BpPYL member, which is BpPYL11. It indicates that BpPYL11 may play a distinct role in functioning. In the same subgroup, OsPYL3 was reported to be induced by multiple abiotic stresses [45]. The ectopic expression of *OsPYL3* in *Arabidopsis* caused increased ABA sensitivity. In Subgroup Ⅱ, the BpPYLs have closer phylogenetic relationships with AtPYLs than OsPYLs. No OsPYLs were classified into Subgroup Ⅲ. This results from the stronger homology between the protein sequences of PYLs in birch and *Arabidopsis*, both dicots, while rice is a monocot. Furthermore, the BpPYL members in Subgroup Ⅲ account for half of all the BpPYLs. The six members have similar gene and motif structures, and all have Motif 4, which does not exist in other BpPYLs. This indicates that the six BpPYLs in Subgroup Ⅲ may have distinct functions from other BpPYLs. The subcellular localization prediction analysis showed that most of the BpPYLs are localized in the cytoplasm or chloroplast, which is consistent with PYL family members in other plant species, like the HaPYLs in sunflower [37] and CsPYLs in tobacco [27]. The sequence structure analysis showed that the *BpPYL*s in the same subgroups have similar exon–intron and motif patterns. This indicates the sequence conservatism during the evolution process. Synteny analysis is an effective tool for the investigation of gene duplication events. Here, two syntenic gene pairs were identified within the BpPYL family members, and 12, 8, 19, and 6 syntenic gene pairs between *BpPYL*s and *AtPYL*s, *OsPYL*s, *PtPYL*s, and *ZmPYL*s, respectively. It is obvious that more gene duplication events exist between the *PYL*s in birch and poplar than in birch and the other three species. This may result from the closer evolutionary relationship between birch and poplar, the only two forest tree species in these five plants. On Chr06 of the birch, *BpPYL5* and *BpPYL6* had the most syntenic gene pairs with PYLs in the other three plant species. This indicates that these two genes were more conserved in the evolution of the PYL gene family.

Various kinds of *cis*-acting elements were identified in the promoters of *BpPYL*s, including stress response (such as stress responsiveness, wound responsiveness, low-temperature responsiveness, salicylic acid responsiveness), phytohormone signaling (such as MeJA responsiveness, gibberellin responsiveness, abscisic acid responsiveness, auxin responsiveness), and growth and development (meristem expression, light responsiveness, anaerobic induction, endosperm expression). This indicates the potential roles of *BpPYL*s in the abiotic stress response and phytohormone signaling processes of birch, which is consistent with the results of GO and KEGG enrichment analyses. In this case, the expressional responses of *BpPYL*s upon ABA, salt, and PEG treatments were investigated by using RT-qPCR. The relative expression levels of most of the *BpPYL*s exhibited significant changes upon these abiotic stresses, which indicates the transcriptional involvement of BpPYLs in the abiotic stress responses of birch.

To date, *PYL*s have been proven to have important functions in various plant species’ abiotic stress responses. In Zhao et al.’s research [46], the overexpression of *PYL9* greatly improved the drought tolerance of the transgenic *Arabidopsis* and rice. In Chen et al.’s research [47], three *PYL*s in cotton, *GhPYL10*, *GhPYL12*, and *GhPYL26*, were upregulated upon drought stress. The overexpression of these three genes in *Arabidopsis* resulted in increased ABA sensitivity, a better growth state, and enhanced drought tolerance. In Zhang et al.’s research [48], a PYL family member in wheat, *TaPYL4*, was identified and characterized. It could specifically interact with TaPP2C2, which is a core module in the ABA signaling of wheat. The expression level of *TaPYL4* showed significant upregulation upon drought stress. The wheat seedlings overexpressed with this gene showed better growth properties and improved drought tolerance. In cotton [49], one of the GhPYL family members, GhPYL8D2, showed a significant expressional change within 12 h after stress treatment. The transgenic cotton overexpressed with *GhPYL8D2* showed increased drought tolerance. This results from the stomata control via the co-regulation of GhPYL8D2 with GhHAI2D, a PP2C family member. *StPYL8-like*, a potato *PYL* gene, shares a high homology with *PYL* genes in other species that are responsive to abiotic stress [50]. It is also responsive to drought and ABA treatment. The transient and stable overexpression of this gene in tobacco enhanced the drought resistance of the transgenic plants. *GhPYL9-5D* and *GhPYR1-3A*, the homologues of *Arabidopsis PYL9* and *PYR1* in cotton, were responsive to ABA treatment [51,52]. The overexpression of these two cotton genes in *Arabidopsis* resulted in ABA hypersensitivity in terms of seed germination, root growth, and stomatal closure. The knock-down of these two genes in cotton reduced the tolerance to PEG-induced drought, salinity, and osmotic stresses of the transgenic plants. In this study, the positive functions of *BpPYL3* and *BpPYL11* were proven with the salt-tolerant yeast transformation method. It indicates promising positive functions of *BpPYL*s in birch tolerance against abiotic stresses. However, the specific functions of *BpPYL*s in birch stress response await further verification with bioinformatic and molecular biological tools. Overall, this study provides an important reference for researches on plant ABA signaling and promising gene resources for the breeding of new varieties with better stress tolerance.

## 4. Materials and Methods

### 4.1. Genome-Wide Identification and Physiochemical Analysis of PYL Members in Birch

The genome and protein data of birch (*Betula platyphylla* Suk.) were downloaded from the phytozome v13.1 database (https://phytozome.jgi.doe.gov/pz/portal.html, accessed on 2 October 2024). With the *PYL* gene sequences from *Arabidopsis thaliana* downloaded from TAIR (The Arabidopsis Information Resource) database (https://www.arabidopsis.org/, accessed on 2 October 2024) as query, the PYL family members in birch were searched in the range of the whole birch genome via BLASTp and the Hidden Markov Model (HMM) file (http://pfam.xfam.org/, PF10604, accessed on 2 October 2024). Afterwards, the potential BpPYL family members were further identified and checked through HMMERv3.1, InterPro (http://www.ebi.ac.uk/interpro/, accessed on 3 October 2024), and CDD (https://www.ncbi.nlm.nih.gov/Structure/cdd/wrpsb.cgi, accessed on 3 October 2024).

The chromosome location information of the *BpPYL* genes on chromosomes was obtained from the phytozome database. These genes were named according to their location order on chromosomes. The results were visualized using Tbtools-II (v1.120).

The basic physiochemical characteristics of BpPYLs, including the length of the genomic sequence, number of amino acids, molecular weight, isoelectric point, hydrophilic mean (GRAVY) score, aliphatic index, chromosome location, and predicted cellular localization were analyzed using ExPASy (http://www.expasy.org/, accessed on 7 October 2024).

### 4.2. Phylogenetic Analysis of PYLs from Birch, Arabidopsis, and Rice

After multiple sequence alignment by BioEdit (version 7.2.5), the sequences of PYLs from birch, *Arabidopsis*, and rice (*Oryza sativa*) were subjected to constructing a phylogenetic tree. The phylogenetic tree (1000 bootstrap replications) was constructed with the neighbor joining (NJ) method using MEGA v7.0 software. The gene IDs of *AtPYL*s and *OsPYL*s are listed in Table S11.

### 4.3. Gene Structure Analysis of BpPYLs

The exon–intron structures of *BpPYL*s were analyzed by using the online software GSDS 2.0 (Gene Structure Display Server: http://gsds.cbi.pku.edu.cn/, accessed on 9 October 2024). The conserved motifs were analyzed via the online tool MEME 5.0 (https://meme-suite.org/meme/, accessed on 9 October 2024). These two results were visualized by using Tbtools-II (v1.120).

### 4.4. Intraspecific and Interspecific Synteny Analysis

Intraspecific synteny analysis was performed using Tbtools-II (v1.120) between *BpPYL*s. For interspecies synteny analysis, the gene sequences of PYLs in poplar (*Populus trichocarpa*) and maize (*Zea mays*) were retrieved from the phytozome database. Interspecific synteny analyses were performed between PYL members from birch and *Arabidopsis*, rice, poplar, and maize. The non-synonymous (Ka) and synonymous (Ks) of the syntenic gene pairs were calculated by using Tbtools-II (v1.120).

### 4.5. cis-Acting Elements Analysis

A length of 2000 bp sequence upstream of each *BpPYL* gene was subjected to the *cis*-acting element analysis by using the PlantCARE online service platform (http://bioinformatics.psb.ugent.be/webtools/plantcare/html/, accessed on 15 October 2024). The result was visualized by using Tbtools-II (v1.120).

### 4.6. GO Enrichment and KEGG Analysis

BpPYL protein sequences were annotated by using EGGNOG-Mapper (http://eggnog-mapper.embl.de/, accessed on 19 October 2024). The GO or KO ID of *BpPYL*s were extracted from the annotated files. The results were visualized by using Tbtools-II (v1.120).

### 4.7. Plant Materials and Treatments

The birch seedlings used in this study were preserved by the State Key Laboratory of Tree Genetics and Breeding, Northeast Forestry University (Harbin). The greenhouse for plant culture was set with 25 °C for temperature and 16 h/8 h for light/dark cycle. The birch seedlings in good and similar growth states were selected for further experimental use. The seedlings for tissue-specific analysis were grown in hydroponic culture (1/2 MS medium with 25 g/L sucrose, 0.02 mg/L NAA, and 0.4 mg IBA) for 60 days. For sampling, seedling roots, stems, and leaves were collected, immediately frozen in liquid nitrogen, and preserved in a refrigerator at −80 °C for further experimental use. Each piece of sample contained tissues from five randomly selected birch seedlings, and three biological replicas were set. For ABA treatment, the 60-day-old birch seedlings were transferred into the hydroponic culture with ABA at a concentration of 100 µM. For salt treatment, the 60-day-old birch seedlings were transferred into the hydroponic culture with NaCl at a concentration of 200 mM. For PEG treatment, the 60-day-old birch seedlings were transferred into the hydroponic culture with PEG at 20% (*W*/*V*). These experiments were set with three biological and technical replicates.

### 4.8. Expression Analysis and Data Processing

The relative expression levels of *BpPYL*s were measured by using RT-qPCR. cDNA was used as template after reverse transcription from RNA extracted by using Mega Pure Plant RNA Kit (Msunflowers Biotech Co., Ltd, Beijing, China). The reverse transcription was performed by using reverse transcription kit (PrimeScriptTM RT reagent Kit, Takara Bio, Kusatsu, Japan). Primers (Table S12) for RT-qPCR were designed according to the downloaded full-length cDNA sequences of BpPYLs, and *18S rRNA* served as the internal reference gene [53]. RT-qPCR was performed with reaction system of 20 µL of THUNDERBIRD Next SYBR qPCR Mix (TOYOBO, Osaka, Japan). Reaction condition: pre-denaturation at 94 °C for 30 s, denaturation at 94 °C for 5 s, renaturation at 58 °C for 15 s, extension at 72 °C for 10 s, steps 2 to 4 performed for 45 cycles, melt curve for 6 s. The reaction was performed on Applied Biosystems 7500 Fast Real-Time PCR System. Each reaction was set with three biological replicates. The relative expression levels of *BpPYL*s were calculated with the 2^−∆∆Ct^ method [54]. Significant difference analysis was calculated by one-way ANOVA followed by Duncan’s multiple range tests.

### 4.9. Salt-Tolerant Yeast Transformation

The coding sequences of *BpPYL3* and *BpPYL11* were cloned into the pYES2-NTB vector via seamless cloning. The salt-tolerant yeast (INVSc1) transformed with recombinant vectors, pYES2-NTB-BpPYL3 and pYES2-NTB-BpPYL11, as well as the negative control (pYES2-NTB), respectively, were cultured on nutrition-deprived yeast media (SD/-Ura) with different concentrations of salt (NaCl). The concentration gradient of salt was set at 0, 0.5, 1.0, 1.5, and 2.0 M. The growth state of yeast indicates salt tolerance.

## 5. Conclusions

In this study, the PYL family members in birch were identified and characterized from a genome-wide range. A total of 12 BpPYL family members were identified and classified into three subgroups according to their phylogenetic relationships. The *BpPYL*s that had closer phylogenetic relationships had more similar exon–intron and motif patterns. Two syntenic gene pairs between *BpPYL*s, as well as 12, 8, 19, and 6 syntenic gene pairs between *BpPYL*s and *AtPYL*s, *OsPYL*s, *PtPYL*s, and *ZmPYL*s, respectively, were identified by synteny analysis. The *cis*-acting elements in the promoters of *BpPYL*s were mainly involved in plant stress response, phytohormone signaling, and growth and development. The results of GO and KEGG enrichment analysis showed that *BpPYL*s were enriched in the pathways mainly related to ABA signaling and cell communication. According to the results of RT-qPCR, most of the *BpPYL*s were responsive to ABA, salt, and PEG treatments. Furthermore, *BpPYL3* and *BpPYL11* improved the salt tolerance of the salt-tolerant yeast in the yeast transformation assay. This study provides a reference for further research on ABA signal transduction and plant abiotic stress responses.

## Figures and Tables

**Table 1:** Physiochemical properties of BpPYLs.

Gene Name	Locus Name Phytozome v13	Genomic Sequence (bp)	Amino Acid No.	Molecular Weight (Da)	Isoelectric Points	GRAVY	Aliphatic Index	Chromosome Location	Cellular Localization
*BpPYL1*	BPChr01G05180	510	170	18,864.43	4.71	−0.203	85.38	Chr01:10459086..10459596 (−)	Cytoplasm
*BpPYL2*	BPChr01G05173	448	129	14,432.06	4.81	−0.444	70.00	Chr01:10462133..10462581 (−)	Chloroplast
*BpPYL3*	BPChr01G05283	510	170	18,882.51	4.60	−0.178	93.55	Chr01:10474336..10474846 (−)	Chloroplast, Cytoplasm
*BpPYL4*	BPChr01G05229	5197	198	22,234.06	5.20	−0.252	77.66	Chr01:10493852..10499049 (−)	Cytoplasm
*BpPYL5*	BPChr06G29476	7591	186	20,892.77	6.65	−0.352	87.84	Chr06:6771830..6779421 (+)	Chloroplast
*BpPYL6*	BPChr06G30627	603	201	21,820.67	6.49	−0.179	87.55	Chr06:36934328..36934931 (+)	Cytoplasm
*BpPYL7*	BPChr07G10003	519	173	19,074.71	5.17	−0.154	82.03	Chr07:24448929..24449448 (−)	Chloroplast, Cytoplasm
*BpPYL8*	BPChr08G24195	3192	163	18,400.18	5.40	−0.091	106.42	Chr08:426381..429573 (−)	Chloroplast, Nucleus
*BpPYL9*	BPChr08G27487	507	169	18,676.02	4.53	−0.319	75.42	Chr08:11935085..11935592 (+)	Cytoplasm
*BpPYL10*	BPChr11G06966	507	169	18,676.02	4.53	−0.319	75.42	Chr11:9495928..9496435 (−)	Cytoplasm
*BpPYL11*	BPChr12G11482	3592	296	32,794.00	6.76	−0.471	77.56	Chr12:238375..241967 (+)	Cytoplasm, Nucleus
*BpPYL12*	BPChr14G02044	624	208	22,530.36	6.18	−0.136	88.36	Chr14:2347659..2348283 (−)	Cytoplasm

**Table 2:** Ka/Ks ratios of *BpPYL*s’ syntenic gene pairs.

Duplicated Gene Pairs	Ka	Ks	Ka/Ks	Effective Length
*BpPYL5*/*BpPYL8*	0.150212211	1.199074478	0.125273462	480
*BpPYL9*/*BpPYL10*	0	0	--	504

## Data Availability

Data are contained within the article and Supplementary Materials.

## Supplementary Materials

The following supporting information can be downloaded at: https://www.mdpi.com/article/10.3390/ijms252413728/s1.
